# Dormancy of Cancer Cells with Suppression of AKT Activity Contributes to Survival in Chronic Hypoxia

**DOI:** 10.1371/journal.pone.0098858

**Published:** 2014-06-06

**Authors:** Hiroko Endo, Hiroaki Okuyama, Masayuki Ohue, Masahiro Inoue

**Affiliations:** 1 Department of Biochemistry, Osaka Medical Center for Cancer and Cardiovascular Diseases, Osaka, Japan; 2 Department of Pathology, Osaka Medical Center for Cancer and Cardiovascular Diseases, Osaka, Japan; 3 Department of Surgery, Osaka Medical Center for Cancer and Cardiovascular Diseases, Osaka, Japan; University of Nebraska Medical Center, United States of America

## Abstract

A hypoxic microenvironment in tumors has been recognized as a cause of malignancy or resistance to various cancer therapies. In contrast to recent progress in understanding the acute response of cancer cells to hypoxia, the characteristics of tumor cells in chronic hypoxia remain elusive. We have identified a pancreatic cancer cell line, AsPC-1, that is exceptionally able to survive for weeks under 1% oxygen conditions while most tested cancer cell lines die after only some days under these conditions. In chronic hypoxia, AsPC-1 cells entered a state of dormancy characterized by no proliferation, no death, and metabolic suppression. They reversibly switched to active status after being placed again in optimal culture conditions. ATP turnover, an indicator of energy demand, was markedly decreased and accompanied by reduced AKT phosphorylation. Forced activation of AKT resulted in increased ATP turnover and massive cell death *in vitro* and a decreased number of dormant cells *in vivo*. In contrast to most cancer cell lines, primary-cultured colorectal cancer cells easily entered the dormant status with AKT suppression under hypoxia combined with growth factor–depleted conditions. Primary colorectal cancer cells in dormancy were resistant to chemotherapy. Thus, the ability to survive in a deteriorated microenvironment by entering into dormancy under chronic hypoxia might be a common property among cancer cells. Targeting the regulatory mechanism inducing this dormant status could provide a new strategy for treating cancer.

## Introduction

The microenvironment within a solid tumor can be highly heterogeneous [1]. Because of incomplete blood vessel networks and the imbalance between proliferation and angiogenesis, the microenvironment in some parts of a solid tumor can be hypoxic and poorly supplied with nutrients [2], [3]. Depending on their microenvironment, cancer cells can show quite different characteristics of cell activity including proliferation, oncogenic pathway activation, and metabolism [4]. Tumor cells in a hypoxic region distant from blood vessels show decreased proliferation [5] and resistance to chemo- or radiotherapy [6], [7]. Recently, using an *in vivo* gene-tagging method, it was demonstrated that tumor cells in hypoxic regions could be the origin of recurrence after radiotherapy [8]. It was also reported that change of gene expression in chronic hypoxia was associated with high recurrence rates in colorectal cancer patients [9]. Investigating the biology of tumor cells in hypoxic conditions might be critical for improving therapeutic efficacy and for eradication of cancer. After the discovery of hypoxia-inducible factor-1α (HIF-1α), transcriptional regulation in response to acute hypoxia has been quite well elucidated [10]. In contrast to the responses of cancer cells to acute hypoxia, however, how cancer cells respond to the important but different condition of chronic hypoxia [11] remains elusive.

PI3K/AKT signaling plays a central role in survival, proliferation, and metabolism in cancer cells [12]. Because of the inappropriate activation of receptor tyrosine kinase (RTK) or PI3K, or loss of PTEN function, constitutive activation of AKT is frequently observed in multiple human cancers [12]. Activated AKT promotes glycolytic or biosynthetic pathways by activating GLUT1, hexokinase 2, or ATP-citrate lyase. One of the downstream molecules of PI3K/AKT is mTOR complex 1 (mTORC1), which promotes protein synthesis and cell growth. Thus, AKT/mTORC1 pathways play important roles for tumor growth and metabolism; however the available materials for biosynthesis are not always abundant in the heterogeneous tumor microenvironment. In the hypoxic region distant from blood vessels, sustained activation of the AKT/mTORC1 pathway could lead to critical depletion of nutrients and energy crisis.

The ability to suppress the basal metabolic rate and enter into a hypometabolic status is a life-saver for many organisms when the energy source such as oxygen and nutrition are limited [13], [14]. Indeed, downregulation of mTORC1 activity in acute hypoxia is widely known [15]–[17], and suppression of mTORC1 is reportedly important for tumor cell survival under stressful conditions [4], [18], [19]. Nevertheless, as noted, the chronic response of cancer cells is less well understood.

One factor hampering improved understanding of the response of cancer cells to chronic hypoxia is the lack of established *in vitro* models. Most studies using cancer cell lines have been carried out within 24 h or up to a few days because most cancer cell lines cannot survive the severe depletion of oxygen or nutrients for a longer period. In the present study, we found that a pancreatic cancer cell line, AsPC-1, can stably survive by entering into an inactive status, dormancy, for weeks under hypoxic conditions. In examining the cellular response to this chronic hypoxia, we found that phosphorylation of AKT was downregulated, enabling AsPC-1 cells to reduce energy demand and survive under stressful conditions. In addition, we found that primary colorectal cancer cells could easily enter dormancy under hypoxic and growth factor–deprived conditions, in which they showed remarkable chemoresistant characters.

## Results

### Survival of cell lines under chronic hypoxia

To investigate the effect of prolonged hypoxia on cancer cells *in vitro*, we examined several pancreatic cancer and colorectal cancer cell lines cultured in 1% oxygen for more than a week to represent the chronic condition, as opposed to the shorter frame of a day or so for the acute condition (Figure S1). Most of the tested cell lines showed no remarkable decrease in cell proliferation and died within 7 days. Thus, it was generally difficult to culture cancer cell lines over a week under hypoxic conditions.

**Figure 1:**
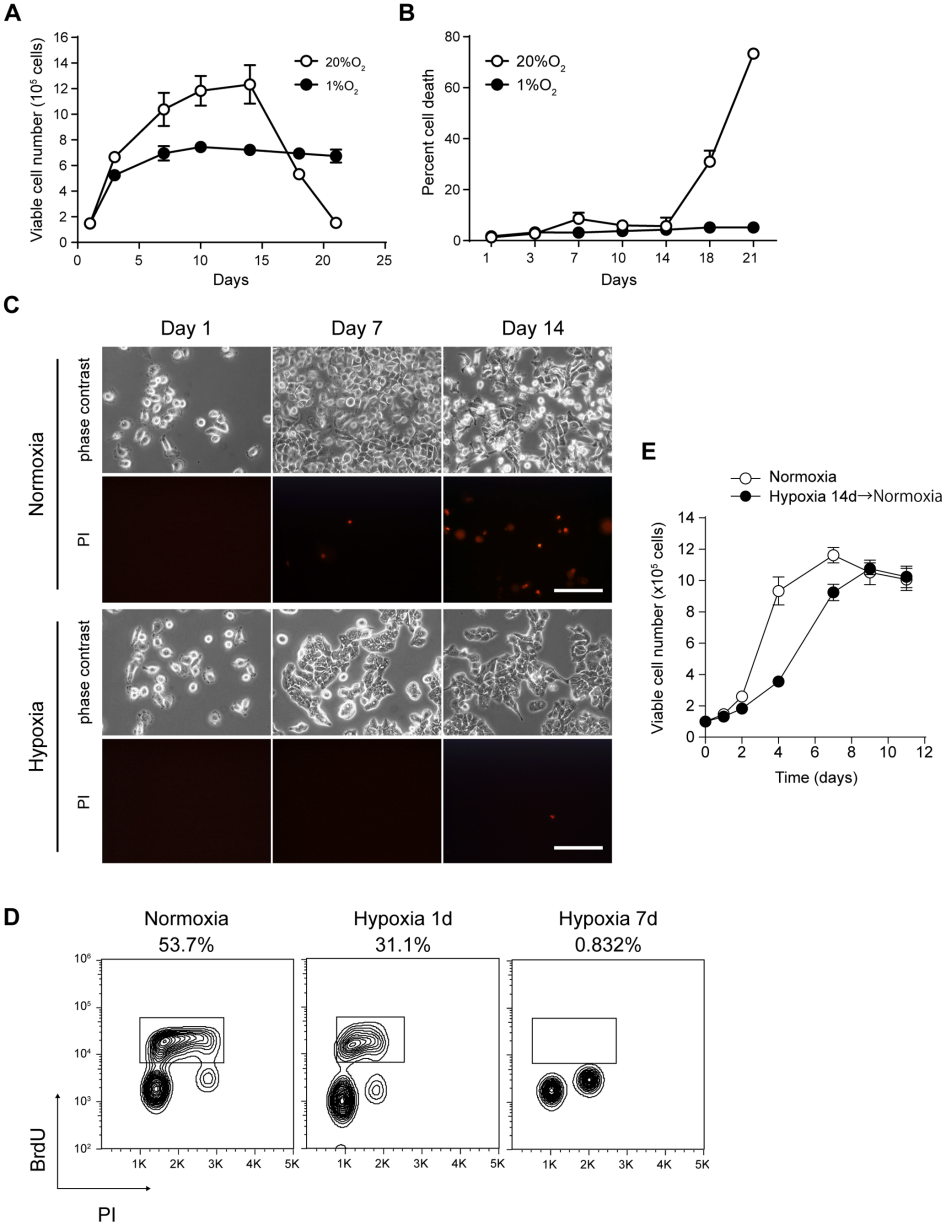
AsPC-1 cells can be in a dormant status in chronic hypoxia. Viable cell number (A) and percent cell death (B) of AsPC-1 cells cultured in normoxia (20% O_2_) or hypoxia (1% O_2_). C) Phase-contrast and PI-stained images of AsPC-1 cells cultured under the indicated conditions. Scale bar  = 50 µm. D) Cell cycle analysis of AsPC-1 cells at day 1 in normoxia or day 1 and day 7 in hypoxia. Cells were pulsed with BrdU for 2 h and analyzed by flow cytometry after staining with anti-BrdU antibody and PI. Percentages of the cells in S phase are indicated. E) Re-growth of AsPC-1 cells in a dormant status. AsPC-1 cells were cultured in hypoxia for 14 days, and the cell counts were monitored after re-seeding in normoxic conditions.

In contrast, AsPC-1, a pancreatic cancer cell line, was exceptionally able to survive under prolonged hypoxic conditions (Figure 1A and C). In the acute phase, AsPC-1 cells in hypoxia grew as well as in normoxic conditions, but the proliferation rate gradually decreased until day 7, and the cell number plateaued around day 10 at less than 80% confluence in the experimental conditions. The cells in normoxia showed massive cell death after 14 days, probably because of the depletion of growth factors or nutrients (Figure 1B and C). In contrast, the cells in hypoxia were viable for more than three weeks without any sign of cell death while the medium was not changed at all. Cell cycle analysis revealed that S-phase cells drastically decreased at day 7 in hypoxia compared with at day 1 in normoxia or hypoxia (Figure 1D). The cells were accumulated in either G0/1 or G2/M phase. This decreased proliferation was reversible; once re-oxygenized and re-plated into the fresh medium, the AsPC-1 cells showed a recovery of the proliferation rate to control levels with a little delay, even after having been cultured for 14 days in hypoxia (Figure 1E). These results indicated that AsPC-1 cells could reversibly enter an inactive status, dormancy, under prolonged hypoxic conditions.

Because most chemotherapy drugs target proliferating cancer cells, cells lingering in dormancy would be resistant to these cytotoxic reagents. Indeed, the AsPC-1 cells cultured in hypoxic conditions were resistant to all three chemo drugs examined (Figure S2A). Both oxygen levels and proliferation rate influence the effectiveness of radiotherapy [20]. AsPC-1 cells in dormant status were more resistant to X-ray irradiation compared to cells in normoxia or in acute hypoxia (Figure S2B). Thus, AsPC-1 cells in dormant status were resistant to conventional chemo- or radio therapy.

### Evaluation of energy metabolism under chronic hypoxia

**Figure 2:**
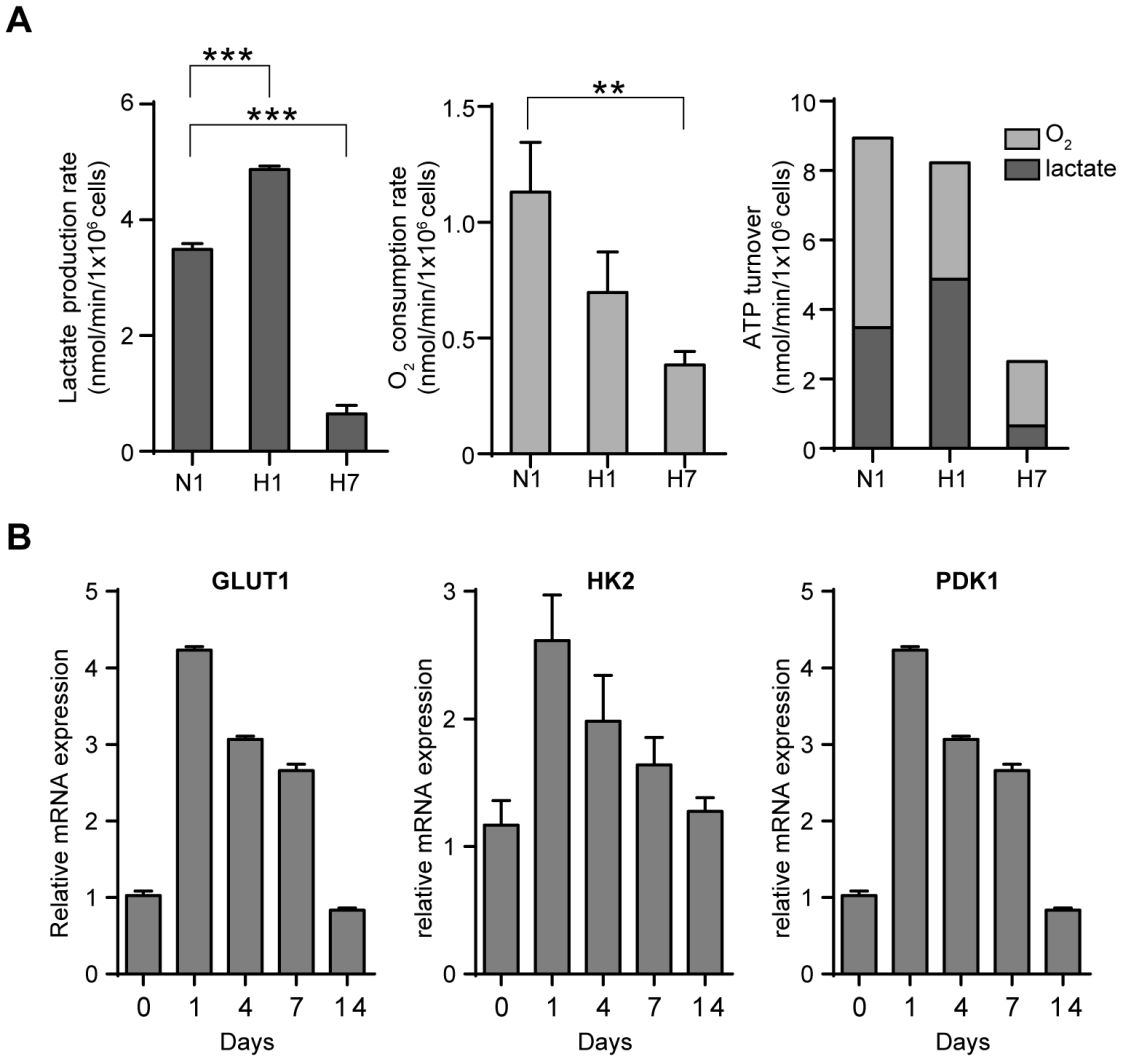
Metabolic processes are suppressed under chronic hypoxia. A) Lactate production rate (left) was calculated from lactate concentration and integral cell number at indicated periods. O_2_ consumption rate (middle) was measured using a Clark type oxygen electrode. ATP turnover (right) was calculated from the lactate production rate and O_2_ consumption rate (dark gray: lactate; light gray: oxygen). N1, normoxia 1 day; H1, hypoxia 1 day; H7, hypoxia 7 days. ** *p*<0.01, ***p<0.001. B) Quantitative RT-PCR of a glucose transporter and glycolytic enzymes in AsPC-1 cells cultured in hypoxia for the indicated days.

We further assessed the status of energy metabolism of the cancer cells in the dormant state. As expected, AsPC-1 cells consumed more glucose and produced more lactate in the acute phase of hypoxia compared with normoxia. In contrast, after 7 days of hypoxia, glucose uptake and lactate production gradually attenuated (Figure 2A, S3A and S3B). The oxygen consumption rate was also decreased under chronic hypoxia (Figure 2A). We calculated the ATP turnover from lactate production rate and oxygen consumption rate (Figure 2A) and found that the ATP turnover decreased under chronic hypoxia. This finding was also supported by a slower decrease in cellular ATP levels after the addition of a cocktail of inhibitors of glycolysis and oxidative phosphorylation (Figure S3C and S3D) [21].

In addition to these assays, we examined the gene expression levels of glycolytic enzymes and transporters (Figure 2B). After exposure to acute hypoxia, the expression of *GLUT1*, *HK2*, and *PDK1* increased. In contrast, the expression levels of these genes decreased in the cancer cells in dormancy. These results were consistent with the decreased glucose uptake in chronic hypoxia (Figure S3A). Taken together, the findings indicated that the dormant cancer cells produced less ATP while consuming less ATP compared with actively dividing cells, suggesting a decreased energy demand. Thus, suppression of the metabolic process is another characteristic of cancer cells in the dormant state.

### Intracellular signaling in cells in the dormant status

**Figure 3:**
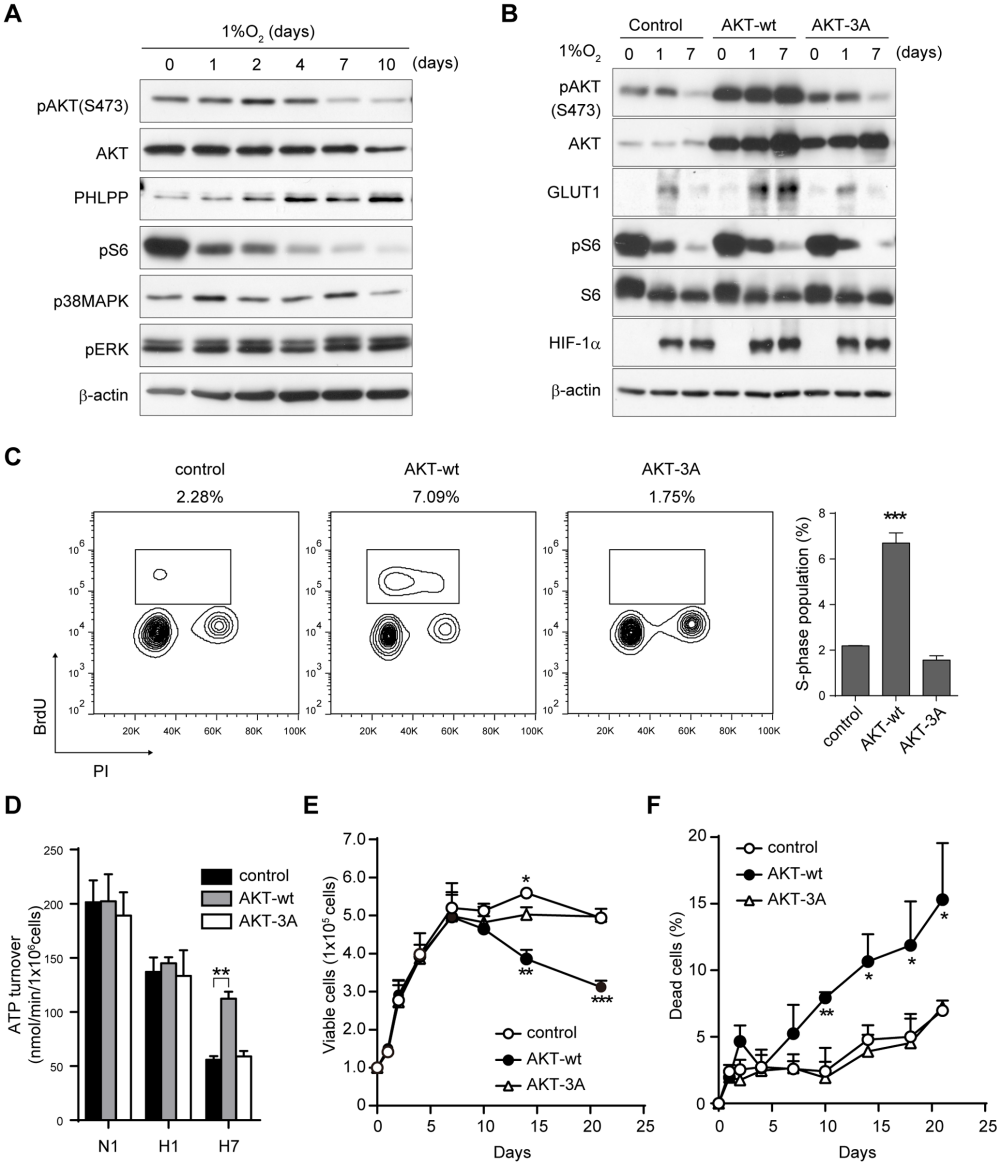
Downregulation of AKT phosphorylation is important for induction of dormant status. A) Immunoblot of the AKT/mTORC1 or ERK/p38 MAPK pathway in AsPC-1 cells cultured in hypoxia. B) Immunoblot of AKT signaling and HIF-1α in AsPC-1 cells expressing vector control, AKT-wt, or AKT-3A (inactive). C) Cell cycle status of the cells at day 7 in hypoxia. Percentages of the cells in S phase are indicated above the plot and in the right graph. D) ATP turnover measured by adding inhibitor cocktail for glycolysis and oxidative phosphorylation. N1, normoxia 1 day; H1, hypoxia 1 day; H7, hypoxia 7 days. E, F) Viable cell number (E) and percent cell death (F) of AsPC-1 cells cultured in hypoxia. *p<0.05, **p<0.01, ***p<0.001.

Next, we investigated intracellular signaling in the dormant-state cancer cells (Figure 3A). Phosphorylation of AKT decreased after 7 days of hypoxia, even under sustained activation of upstream RTKs (Figure S4A). Phosphorylation of S6, a downstream molecule of mTORC1, was decreased from an earlier time point, as was reported previously [15], [16]. Upregulation of phospho-p38MAPK and downregulation of phospho-ERK have been reported to be a molecular switch for induction of dormant status in several cancer cell lines [22], [23], but we observed little change in their levels, and phospho-p38MAPK was rather decreased in chronic hypoxia. Phosphorylation of eIF2α, which subsequently downregulates global protein synthesis [24], was upregulated in acute hypoxia but reduced in chronic hypoxia (Figure S4A), suggesting a critical difference in cell response under these two conditions. The levels of PHLPP, an AKT Ser473-specific phosphatase [25], [26], were reciprocally increased with AKT de-phosphorylation (Figure 3A).

Because the decreased AKT phosphorylation coincided with induction of the dormant state, we examined the functional role of AKT phosphorylation in dormancy. We transduced three AKT constructs into AsPC-1 cells: wt-AKT, AKT-3A (inactive form of AKT), and AKT-mΔPH (constitutive active form of AKT) [27]. When wt-AKT or AKT-mΔPH was overexpressed, AKT phosphorylation was sustained even in chronic hypoxia (Figure 3B, S4B). In control cells, the expression of GLUT1 increased in acute hypoxia but decreased in prolonged hypoxia, again highlighting an opposing response under the two conditions. In contrast, in wt-AKT–overexpressing cells, GLUT1 levels remained high even in chronic hypoxia (Figure 3B), accompanied by continuous glucose consumption (Figure S4C). On the other hand, S6 phosphorylation in chronic hypoxia was decreased even in wt-AKT–overexpressing cells (Figure 3B). Cell cycle analysis revealed that wt-AKT–overexpressing cells in chronic hypoxia showed a higher proliferation rate compared to control cells (Figure 3C, Figure S4D). In AsPC-1 cells expressing control vector or inactive AKT-3A, ATP turnover decreased after 7 days of culture in hypoxia (Figure 3D); however, wt-AKT–expressing cells sustained high ATP turnover even in chronic hypoxia. These results indicated that sustained activation of AKT signaling inhibited AsPC-1 cells from entering into a dormant state in chronic hypoxia, implying a role for AKT suppression in entering dormancy. The viable cell number decreased whereas the death rate increased after 14 days in wt-AKT–expressing cells (Figure 3E and F). AKT-mΔPH–expressing cells showed massive cell death at early time points under hypoxic conditions (Figure S4E and S4F). Finally, when PTEN, a phosphatase of PI3K, was knocked down, a slight increase in AKT phosphorylation was observed, accompanied by an increase in cell death in chronic hypoxia (Figure S5). Taken together, these findings indicate that suppression of AKT phosphorylation is functional in the survival of the cancer cells in the dormant status.

### HIF-1α partially contributes to the induction of dormant status in chronic hypoxia

Next, we examined the contribution of HIF in inactive cancer cells (Figure S6). Protein levels of HIF-1α were increased after exposure to hypoxia and maintained until day 7 (Figure S6D). When HIF-1α levels were forcibly decreased by shRNA (Figure S6A), the viable cell number decreased after 7 days whereas the death rate increased (Figure S6B and S6C), suggesting that sustained HIF-1α also contributed to cell survival under chronic hypoxia. Knockdown of HIF-1α had little effect on AKT/mTORC1 signaling and somewhat increased pS6 levels (Figure S6D), but overexpression of AKT had no effect on induction of HIF-1α (Figure 3B). These results indicated that HIF-1α and AKT independently regulated the dormant status in chronic hypoxia.

### Alteration of dormant status *in vivo* by forced activation of AKT

**Figure 4:**
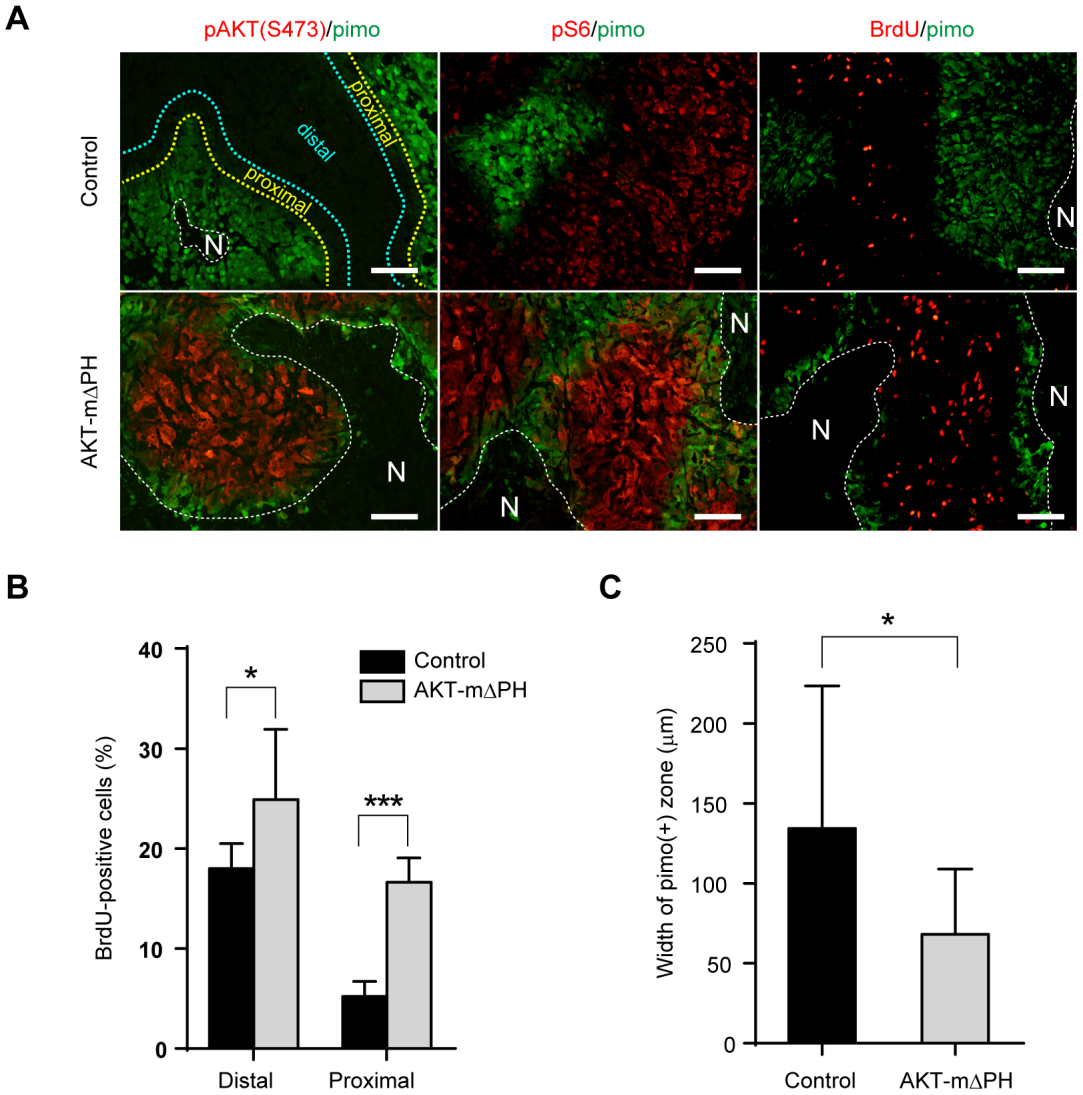
Forced activation of AKT reduces the inactive zone *in vivo*. A) Immunohistochemistry of xenotumors of AsPC-1 cells expressing control vector (upper) or AKT-mΔPH (lower). N, necrosis; scale bar  = 100 µm. B) Percent of BrdU-positive cells in the area distal or proximal to pimonidazole-positive zone. C) Width of pimonidazole-positive zone in tumors from vector or AKT-mΔPH; *p<0.05, ***p<0.001.

We then examined the characteristics of tumors derived from AKT-mΔPH–expressing AsPC-1 cells *in vivo* (Figure 4). Phosphorylation of AKT was detectable only in the AKT-mΔPH tumors but not in the control tumors (Figure 4A). We observed downregulation of S6 phosphorylation and bromodeoxyuridine (BrdU) uptake in the pimonidazole-positive area and its proximal zone in control tumors, consistent with our previous report using another cancer cell line [4]. These areas suggest the existence of a zone of hypoxia-induced dormancy *in vivo*. In contrast, AKT-mΔPH–expressing tumors rarely contained the dormant zone in the pimonidazole-proximal region. The pS6- or BrdU-positive cells were observed even at the boundary of necrosis (Figure 4A).

We further quantified the BrdU-positive cells in the areas proximal or distal to the pimonidazole-positive zone (Figure 4B). The percentage of BrdU-positive cells was remarkably decreased in the area proximal to the pimonidazole region compared to the distal area in control tumors. In contrast, AKT-mΔPH–expressing tumors contained high levels of proliferating cells even in the proximal area. Furthermore, in AKT-mΔPH tumors, the area of the pimonidazole-positive cells was decreased relative to control tumors (Figure 4C), indicating that the AKT-mΔPH cells could not enter into a dormant status *in vivo* and were more prone to death under hypoxic conditions.

### Induction of dormant status in primary colorectal cancer cells

**Figure 5:**
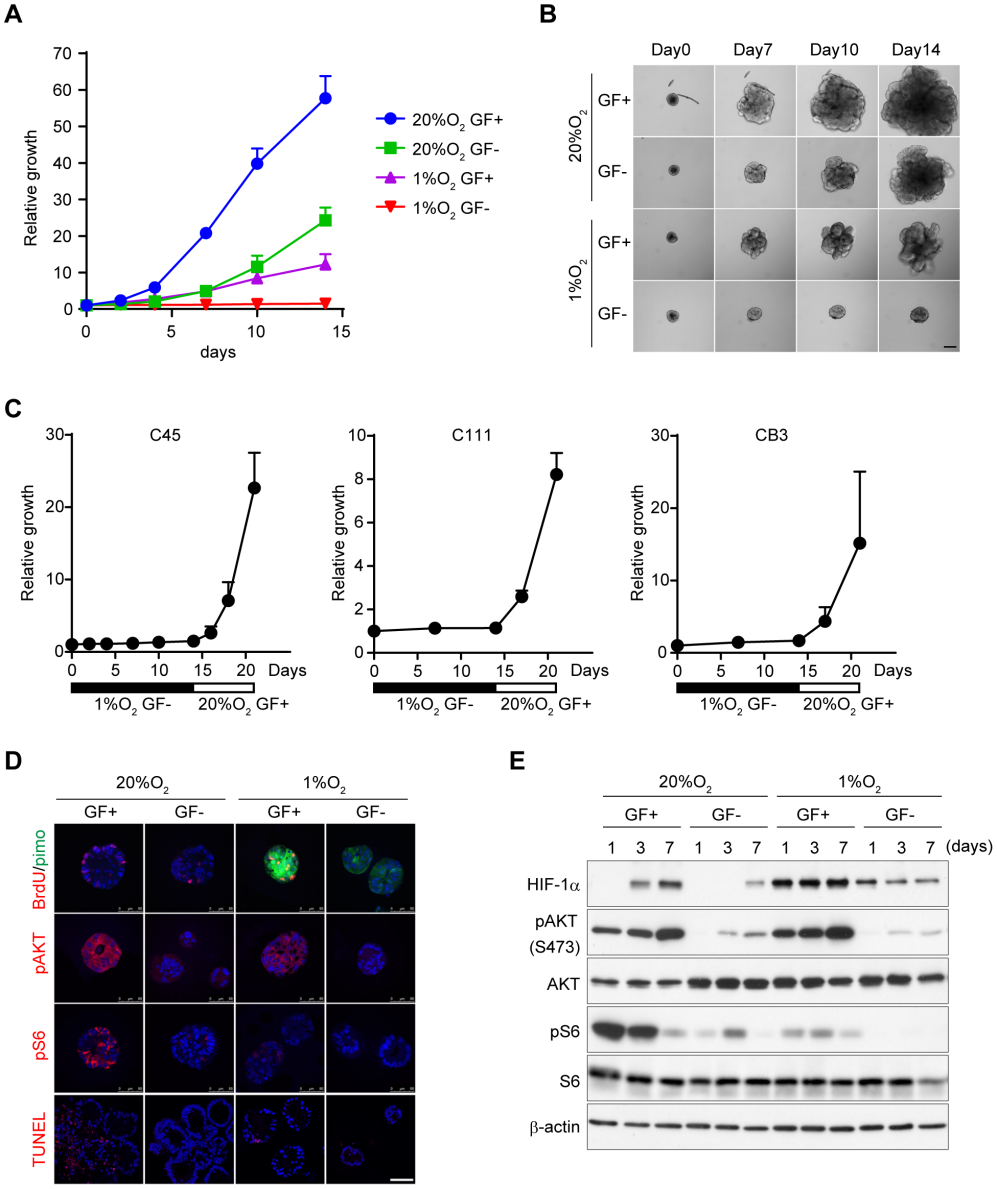
Downregulation of AKT phosphorylation is necessary for induction of dormant status in primary colorectal cancer. A) CTOS growth was measured by size relative to day 0. C45 CTOS samples were cultured in medium with (GF+) or without (GF−) growth factors. B) Representative images of C45 CTOS cultured in indicated conditions. Scale bar  = 100 µm. C) Regrowth of CTOS in dormant status after re-oxygenation and exposure to growth factor–containing medium. D) Immunohistochemistry of C45 CTOS cultured in indicated conditions for 1 day. TUNEL staining was at day 14. Scale bar  = 50 µm. E) Immunoblot of AKT/mTORC1 signaling and HIF-1α in C45 CTOS cultured in indicated conditions.

Next, we examined whether induction of dormant status under chronic hypoxia was also observed in primary cultured cancer cells. We recently established a novel primary culture system, CTOS (cancer tissue–originated spheroid), in colorectal, lung, and urothelial cancer [28]–[30]. We prepared CTOS samples from colorectal cancer patients and cultured them *in vitro* (Figure 5A and B). CTOS growth was completely inhibited under a combination of hypoxia and growth factor–deprived conditions, although hypoxia alone was not sufficient to eliminate the growth. We tested CTOS from three colorectal cancer patients, and all samples re-grew well immediately after re-exposure to oxygen and growth factor–containing medium (Figure 5C). Thus, the induction of dormancy was not restricted to AsPC-1 cells but also was observed in primary cancer cells.

Furthermore, we examined intracellular signaling in CTOS by immunohistochemistry and immunoblot (Figure 5D and E). Hypoxia combined with growth factor depletion completely blocked AKT signaling. These results were consistent with CTOS growth (Figure 5A). We also measured oxygen and glucose consumption in CTOS samples (Figure S7). Hypoxia and growth factor–deprived conditions severely attenuated these metabolic processes, as also was observed in AsPC-1 cells.

**Figure 6:**
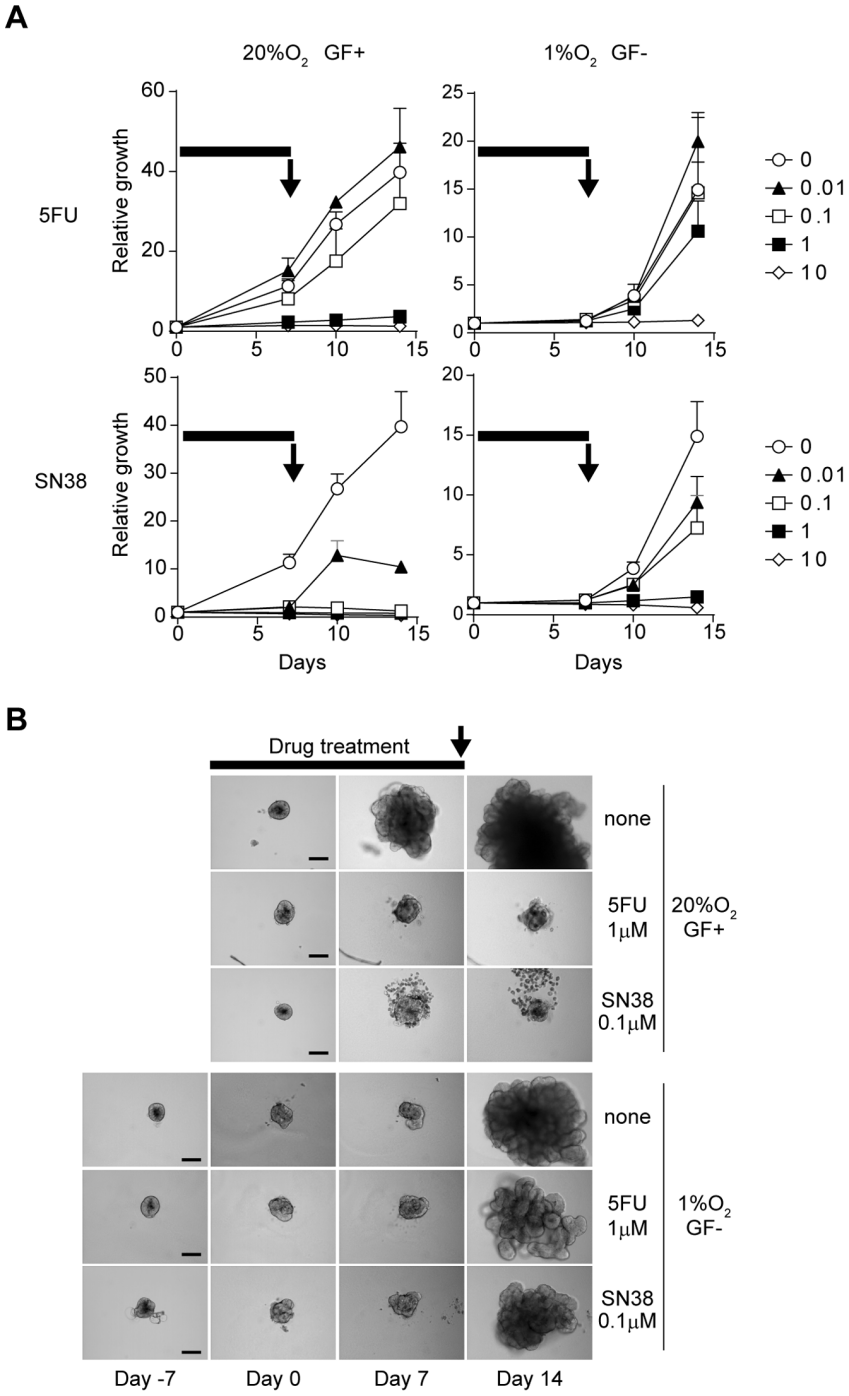
Primary colorectal cancer in dormant status is resistant to chemotherapy. A) CTOS samples were cultured in medium with (GF+) or without (GF−) growth factors, and under 20% O_2_ or 1% O_2_ conditions. 5FU or SN38 were added to medium and treated for 7 days (indicated by black bars). At day 7, medium was changed to fresh StemPro hESC containing growth factors (black arrows), and CTOS samples were allowed to regrow under 20% O_2_. B) Representative images of CTOS samples in (A). Scale bar  =  100 µm.

We then examined the chemo-sensitivity of CTOS in dormant status (Figure 6). CTOS samples were pre-cultured in hypoxia and growth factor–deprived conditions for 7 days. After that, they were exposed to 5FU or SN38, the active metabolite of irinotecan, for 7 days, followed by washing and culturing in fresh StemPro hESC. The CTOS samples in the dormant status showed regrowth after being returned to optimal culture conditions at a 10-fold higher dose than the CTOS samples in the active status (Figure 6A and B). These results indicated that cancer cells in the dormant state were more resistant to chemotherapies than those in an active state.

## Discussion

We demonstrated in the present study that one cancer cell line and primary colorectal cancer cells alter proliferation and metabolic status under prolonged hypoxic conditions. Under chronic hypoxia, the cells could enter into a dormant state involving four characteristics: 1) no proliferation, 2) no death, 3) metabolic suppression, and 4) recovery to active status after restoration of optimal culture conditions.

AKT is a key molecule regulating cell proliferation, survival, and metabolism. It is widely accepted that activation of AKT contributes to cell survival and drug resistance in acute hypoxia [1], [31]–[33]. In contrast, as we demonstrated here, in chronic hypoxia, suppression of AKT activity is necessary for induction of dormancy and survival of the cancer cells. Because the supply of both oxygen and nutrients would be restricted in an area away from the blood vessels in a malignant tumor, preserving the energy source by decreasing energy demand might be a strategy of cancer cells to survive in a chronically deteriorated microenvironment.

The mechanism by which AKT activity is suppressed in chronic hypoxia remains an open question. Possible candidates include 1) inactivation of upstream kinases such as RTKs and PI3K, 2) feedback loop by S6K activation, or 3) activation of phosphatases such as PTEN and PHLPP. The first two possibilities are unlikely given the following findings in the current work: 1) phosphorylation of ERBB family RTKs and MET remained high even in chronic hypoxia (Figure S4A), and 2) mTORC1 activity is suppressed both in acute and chronic hypoxia (Figure 3A). The third possibility is supported by our findings that the levels of PHLPP increased in parallel with AKT inactivation (Figure 3A) and that forced suppression of PTEN promoted death in chronic hypoxia (Figure S5B). Further study will elucidate the precise mechanism.

In the stress response in hypoxia, downregulation of mTOR activity, the unfolded protein response, and transcriptional activation of HIF have been well studied [10], [34]. REDD1 suppresses mTORC1 activity through TSC1/2 in hypoxia, resulting in inhibition of Cap-dependent mRNA translation [15], [16]. In the unfolded protein response, an ER resident kinase, PERK, phosphorylates eIF2α and suppresses mRNA translation [24]. Because protein synthesis is an energy-consuming process, the pathways suppressing it might play role in the induction of dormancy under chronic hypoxic conditions. Indeed, we observed suppression of mTORC1 activity and an increased phosphorylation of eIF2α in acute hypoxia (Figure 3A, Figure S4A). Because cell proliferation and metabolism were sustained in acute hypoxia, they might be necessary but not enough to induce dormancy in chronic hypoxia. HIF-1α and HIF-2α are key transcription factors that regulate the hypoxic response [10]. In acute hypoxia, HIF proteins are stabilized and activate the transcription of various downstream genes. On the other hand, in prolonged hypoxia, HIF proteins are reportedly downregulated by feedback mechanisms. In our results, however, expression of HIF-1α was sustained in dormant status during prolonged hypoxia (Figure S6D), and knockdown of HIF-1α resulted in increased cell death in chronic hypoxia (Figure S6A and S6B), indicating that sustained expression of HIF-1α was also necessary for survival of AsPC-1 cells under chronic hypoxia.

Although uncontrolled proliferation is a hallmark of cancer, human tumors as well as xenografts of human tumors exhibit proliferative indices as low as 20% [35]. These non-proliferating cells in tumors have sometimes been referred to as quiescent cells. Recently, the cancer stem cell theory, in which tumors are assumed to be maintained by their own stem cells, has been intensively tested [36]. Because quiescence is a hallmark of normal adult stem cells, quiescent cancer stem cells have been speculated, but their existence in human solid tumors has not been directly explored [36]. In adult stem cells, quiescence is defined as a cell cycle phase, G0 [37], while AsPC-1 cells in the dormant status were present in the current work in both G1/G0 and G2/M phases (Figure 1C). The dormant status in this study might differ from quiescence, but the associations remain open questions.

Tumor dormancy has been studied as the condition in which tumors remain asymptomatic for a long period, years in some cases [37], [38]. Two models exist for tumor dormancy, tumor mass dormancy and tumor cell dormancy. The former assumes a state of equilibrium between cell proliferation and cell death, while in the latter case, tumor cells enter cell cycle arrest and remain quiescent. The hypoxia-induced dormancy identified in this study, resulting from chronic rather than acute hypoxia, might share features with the latter, but the key molecules identified here differ. In an epidermoid carcinoma cell line, for example, the balance of phospho-ERK and phospho-p38 MAPK has been reported to be the molecular switch for induction of tumor cell dormancy [39]. Signaling from an unfavorable microenvironment or cell surface receptors upregulates phospho-p38 MAPK, and cells then enter G1/G0 phase. In contrast, the key event for induction of inactive status in the current work was downregulation of pAKT but not activation of p38 MAPK (Figure 3A).

Among the five cell lines we tested, only AsPC-1 cells could enter the dormant status under conditions of chronic hypoxia (Figure 1 and S1). In contrast, all three primary colorectal cancer cells examined could enter into a dormant status under a combination of hypoxia and growth factor depletion (Figure 5). Because cancer cell lines are selected cells with a growth advantage under culture conditions with high oxygen, nutrition, and growth factors, they might have lost the ability to suppress proliferation under deteriorated conditions. Thus, CTOS could provide a platform to investigate the dormancy of cancer cells. Although dormant cancer cells do not directly contribute to tumor growth, they can be a reservoir and a source of tumorigenic cells and chemoresistance. The mechanism of pAKT downregulation under chronic hypoxia could be a target of new drugs designed to overcome therapeutic resistance.

## Material and Methods

### Ethics Statement

Preparation and culture of primary colorectal cancer from patients were approved by the Ethics Committee, Osaka Medical Center for Cancer and Cardiovascular Diseases (OMCCCD), and surgical specimens were obtained upon written informed consent. Animal studies were approved by the OMCCCD Institutional Animal Care and Use Committee, and performed in compliance with the institutional guidelines.

### Cells and cell culture

A pancreatic cancer cell line, AsPC-1, was obtained from the American Type Culture Collection (ATCC, Rockville, MD). AsPC-1 was cultured in RPMI 1640 medium supplemented with 10% fetal bovine serum. Hypoxic culture was achieved by incubating cells with 1% O_2_ and 5% CO_2_ in a Multigas Incubator (ASTEC, Fukuoka Japan). Cells were seeded at a density of 1×10^5^ cells per 35-mm dish for counting cell number and 2.5×10^5^ cells per 60-mm dish for RT-PCR and Western blotting. Cell viability was assessed by the trypan blue dye exclusion assay or propidium iodide (PI) staining.

### Primary culture of colorectal cancer

Preparation and culture of primary colorectal cancer cells were performed using the CTOS method [28]. Briefly, surgical samples or xenograft tumors of NOD/SCID mice were partially digested with Liberase DH (Roche, Mannheim, Germany) and filtered through cell strainers. Fragments on the 100-µm or 40-µm cell strainer (BD Falcon, Franklin Lakes, NJ) were collected and cultured in StemPro hESC (GIBCO). For hypoxic culture, CTOS samples were embedded in BD Matrigel Matrix Growth Factor Reduced (GFR) (BD Biosciences, San Jose, CA) and cultured in StemPro hESC or basal medium (DMEM F12/Glutamax, 0.1 mM 2-mercapthoethanol, 2% bovine serum albumin). For the chemosensitivity assay during the inactive period, CTOS samples were pre-cultured in basal medium under 1% O_2_ conditions for 7 days, and then treated with 5-FU or SN38. After 7 days of exposure to these drugs, the CTOS samples were re-oxygenated, and the culture medium was changed to fresh StemPro hESC.

### Cell cycle analysis

AsPC-1 cells were cultured for the indicated time periods and labeled with 10 µM BrdU for the last one hour of each treatment. The cells were stained with 5 µg/mL of PI and anti-BrdU antibody (BD Pharmingen). The cells were analyzed using an Attune Acoustic Focusing Cytometer (Life Technologies, Carlsbad, CA).

### Measurement of glucose and lactate concentration

Glucose concentration in culture medium was measured using Glucose Test 2 (Wako Pure Chemical, Osaka, Japan). Lactate was measured using the F-Kit L-Lactic acid (Roche, Darmstadt, Germany).

### Measurement of oxygen consumption

Dissolved oxygen was measured using a Clark-type oxygen electrode (Model 203, Instech Laboratories). AsPC-1 cells were collected and suspended in RPMI 1640 medium, which was equilibrated for 20% or 1% O_2_ concentration. Oxygen consumption rate was calculated as previously described [40].

### ATP turnover estimation

ATP turnover was estimated by two methods. First, ATP equivalents were calculated with the assumption of a P/O ratio of 2.41 (Brand 1994 Biochemist) and one mole of ATP per mole of lactic acid produced. The second method involved assessing the attenuation rate of intracellular ATP levels after blockade of ATP synthesis [21]. AsPC-1 cells were seeded at a density of 1×10^4^ cells per well in 96-well black plates (Corning, Rochester, NY) and cultured under normoxic or hypoxic conditions. Cells were treated with a cocktail of 1 µM Antimycin A, 5 mM KCN, and 2.5 mM 2-deoxyglucose, and the cellular ATP levels were measured at each time point using the Cell Titer-Glo Luminescent Cell Viability Assay (Promega, Madison, WI). ATP turnover was calculated from the slope of the reduction curve of cellular ATP.

### Quantitative RT-PCR

Total RNA was isolated from the cells using the RNeasy Mini kit (Qiagen). One microgram of total RNA was reverse transcribed to obtain cDNA using Superscript III (Invitrogen) according to the manufacturer’s protocol. The quantitative PCR reactions were performed with the StepOne Real Time PCR System (Life Technologies, Carlsbad, CA) using Fast SYBR Green Master MIX. The primer sequences are given in Table S1.

### Western blot

Western blot was performed as previously described [18]. Primary antibodies used against phospho-AKT (Ser473) (clone D9E), AKT (clone 40D4), phospho-S6 (Ser235/236), S6, phospho-p38 MAPK (clone D3F9), and phospho-ERK (Thr202/Tyr204) (clone D13.14.4E) were obtained from Cell Signaling Technologies; HIF-1α (clone 54) from BD Transduction Laboratories (San Jose, CA); PHLPP and GLUT1 from Abcam (Cambridge, UK); PTEN from Santa Cruz (Santa Cruz, CA); and β-actin from SIGMA. For CTOS, Matrigel GFR-embedded CTOS were retrieved from the gel by incubating in PBS-EDTA (5 mM EDTA, 1 mM NaVO_4_, 1.5 mM NaF) for 1 h at 4°C before cell lysis [41].

### Immunohistochemistry

Immunohistochemistry was performed on formalin-fixed, paraffin-embedded tumor sections. Sections were dewaxed, rehydrated, and subjected to antigen retrieval by autoclave incubation in citrate buffer (pH 6.0). The primary antibodies against phosphor-S6 (Ser235/236) and phosphor-AKT (Ser473) were obtained from Cell Signaling Technologies (Danvers, MA) and BrdU from BD Pharmingen (San Jose, CA). After secondary antibody incubation, sections were visualized with a fluorescence method. For detection of the hypoxic region, fluorescein isothiocyanate–labeled hypoxyprobe-1 monoclonal antibody (NPI, Belmont, MA) was used. Apoptosis was detected with the ApopTag Red In Situ Apoptosis Detection Kit (CEMICON, Temecula, CA), and image analysis was performed with software cellSens (Olympus, Tokyo, Japan). The percentage of BrdU-positive cells was measured by Lumina Vision (Mitani Corp., Tokyo, Japan) in two regions of interest: a proximal region (within 50 µm of the pimonidazole-positive zone) and a distal region (more than 50 µm from the pimonidazole-positive zone).

### Establishment of stable expression cell lines

Plasmids of pCS2/hAKT-wt, pCS2/hAKT-3A (inactive form of AKT, in which the amino acid residues of Lys179, Thr308, and Ser473 were replaced with alanine), and pCS2/hAKT-mΔPH (constitutive active form of AKT, in which the pleckstrin homology domain was removed and myristoylation signal was added) were generous gifts from Dr. Goto, Tokyo University [27]. They were transferred into the pMXspuro vector and transduced into AsPC-1 cells by retro-viral infection. The cells permanently expressing these genes were selected by puromycin.

### Animal studies

AsPC-1 cells expressing control vector or AKT-mΔPH were transplanted into the flanks of nude mice. To detect proliferative or hypoxic cells in tumors, BrdU or pimonidazole was injected intraperitoneally into the mice 2 h before sacrifice.

### Statistical analysis

Statistical analysis was carried out with GraphPad Prism 4 (GraphPad Software, San Diego, CA). The statistical significance was tested using unpaired *t*-tests. A value of *p*<0.05 was considered to indicate statistical significance.

## Supporting Information

Figure S1 **Common cancer cell lines could not be in a dormant status in hypoxia.** Colorectal cancer cell lines (DLD-1, COLO320) and pancreatic cancer cell lines (MIA PaCa-2, PANC-1) were cultured in 20% O_2_ or 1% O_2_. Viable cell number (left panel) and percent cell death (right panel) were measured by trypan blue dye exclusion.
(TIF)

Figure S2 **AsPC-1 cells in a dormant status are resistant to chemo- or radiotherapy.** A) AsPC-1 cells cultured in 20% O_2_ or 1% O_2_ for 8 d were treated with indicated chemo drugs for 3 days. B) AsPC-1 cells were cultured in normoxia, hypoxia 6 h, or hypoxia 7 d and irradiated with X-ray at the indicated dose. The irradiated cells were seeded at clonal density, and the survival fraction ( =  number of colony/seeded cells) was calculated.
(TIF)

Figure S3 **ATP turnover is decreased in chronic hypoxia.** Glucose (A) or lactate (B) concentration in conditioned medium of AsPC-1 cells cultured in hypoxia. C) AsPC-1 cells were cultured in a 96-well plate for the indicated periods. Inhibitor cocktail for OXPHOS (KCN, Antimycin A) and glycolysis (2-deoxyglucose) was added at the same time, and the decrease in cellular ATP was measured by Cell Titer Glo. D) ATP turnover was calculated from the slope of the ATP attenuation curve. N1, normoxia 1 day; H1, hypoxia 1 day; H7, hypoxia 7 days.
(TIF)

Figure S4 **Downregulation of AKT phosphorylation is important for induction of dormant status.** A) Western blot of phospho-RTKs or peIF2α in AsPC-1 cells cultured in hypoxia for indicated days. B) Western blot of AKT signaling in AsPC-1 cells expressing control vector or AKT-mΔPH (constitutive active). Black arrowhead indicates endogenous AKT, and white arrowhead indicates AKT-mΔPH. C) Glucose concentration in conditioned medium of AsPC-1 cells cultured in hypoxia. D) Percentage of cells in S phase at normoxia day 1 (N1), hypoxia day 1 (H1), or hypoxia 7 days (H7). Viable cell number (E) and percent cell death (F) of AsPC-1 cells expressing control vector or AKT-mΔPH cultured in hypoxia. *p<0.05, **p<0.01, ***p<0.001, ****p<0.0001.
(TIF)

Figure S5 **PTEN partially works in the dormant status of AsPC-1 cells.** A, B) Viable cell number (A) or percent cell death (B) of AsPC-1 cells expressing control vector or shRNA for PTEN (PTENi) cultured in hypoxia; **p<0.01. C) Western blot of AKT signaling in AsPC-1 cells expressing PTENi.
(TIF)

Figure S6 **HIF-1α partially contributes to the induction of dormant status in chronic hypoxia.** A) Western blot of HIF-1α in AsPC-1 cells expressing control vector or two different clones of shRNA for HIF-1α (shHIF#1 or #3). B) Percent cell death of AsPC-1 cells expressing shHIF#1 or #3 cultured in hypoxia for 10 days. C) Viable cell number of AsPC-1 cells expressing shHIF#3 cultured in hypoxia. D) Western blot of AKT/mTORC1 signaling in AsPC-1 cells expressing shHIF#3. *p<0.05, **p<0.01.
(TIF)

Figure S7 **Metabolic processes are suppressed under dormant status in primary colorectal cancer cells.** A) Oxygen consumption of C45 CTOS cultured in indicated conditions measured by CRAS; *p<0.05, **p<0.01, ***p<0.001. B) Glucose concentration in culture medium in indicated conditions.
(TIF)

Materials and Methods S1 (DOC)

Table S1 **Primer sequence for real time PCR.**
(XLS)
