# Supplementary figures and images for: Dormancy of Cancer Cells with Suppression of AKT Activity Contributes to Survival in Chronic Hypoxia
Source: PLoS One. 2014 Jun 6;9(6):e98858. doi: 10.1371/journal.pone.0098858 (PMC4048292; doi:10.1371/journal.pone.0098858)

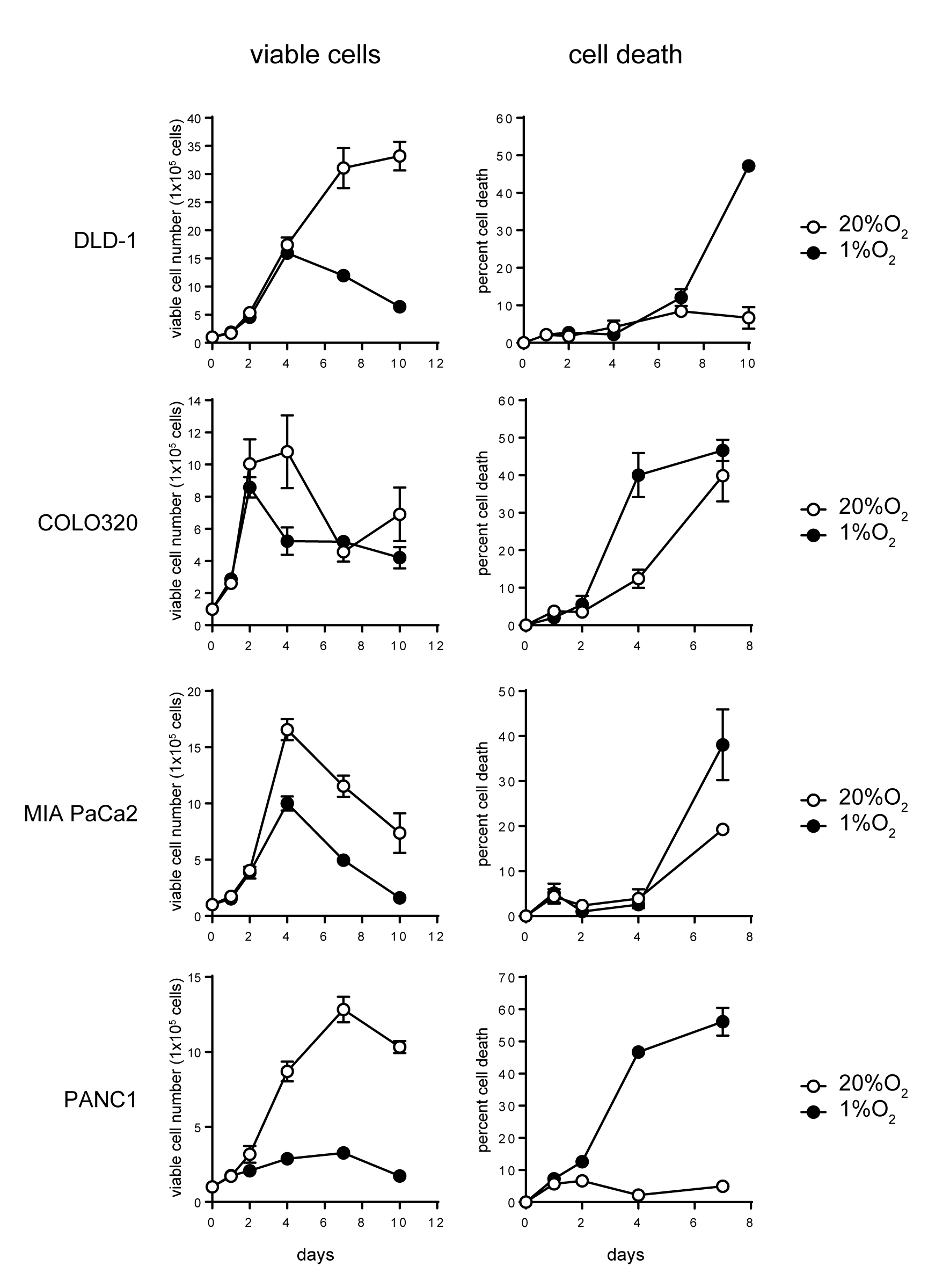

Supplement: Figure S1 — Common cancer cell lines could not be in a dormant status in hypoxia. Colorectal cancer cell lines (DLD-1, COLO320) and pancreatic cancer cell lines (MIA PaCa-2, PANC-1) were cultured in 20% O2 or 1% O2. Viable cell number (left panel) and percent cell death (right panel) were measured by trypan blue dye exclusion. (TIF) [file pone.0098858.s001.tif]

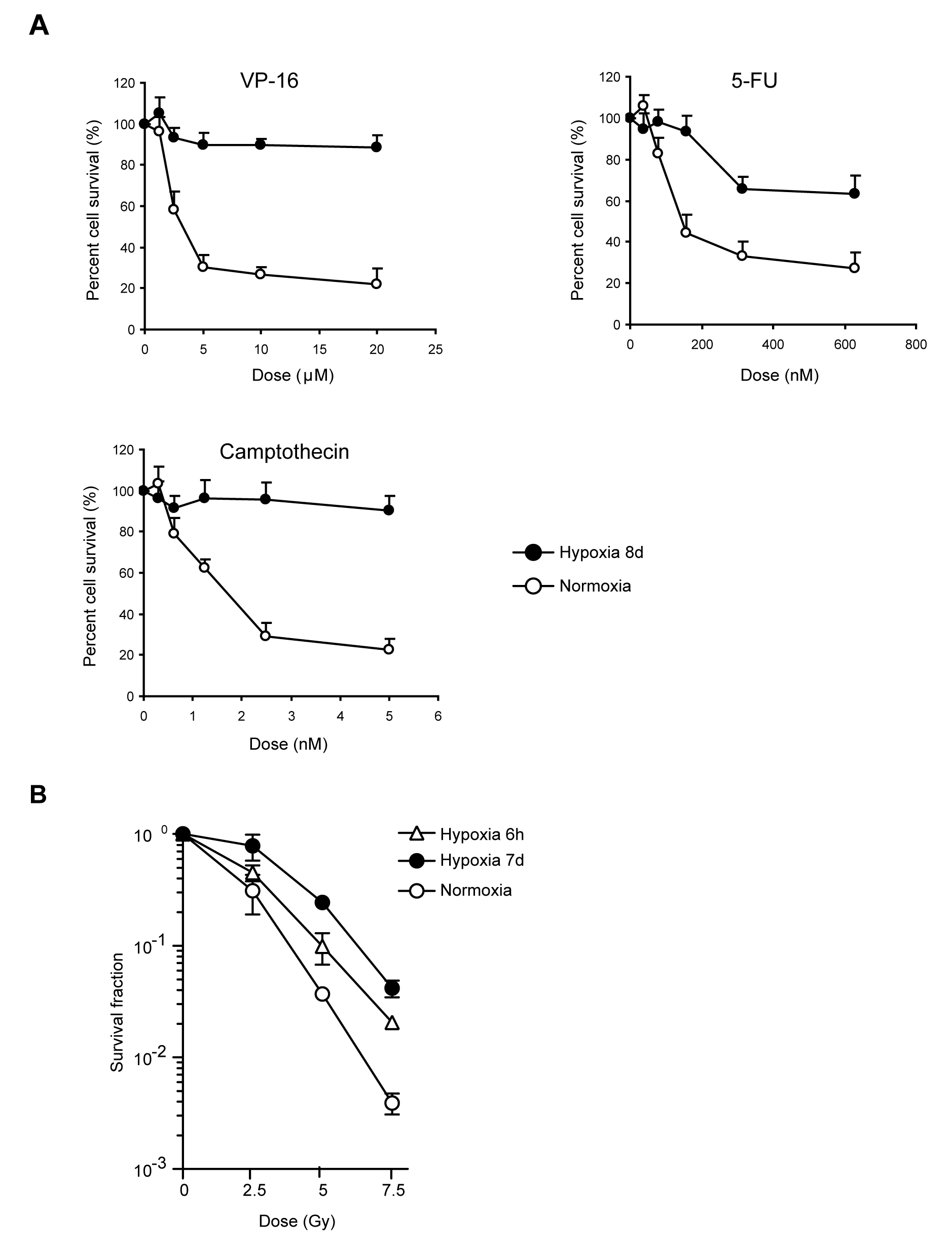

Supplement: Figure S2 — AsPC-1 cells in a dormant status are resistant to chemo- or radiotherapy. A) AsPC-1 cells cultured in 20% O2 or 1% O2 for 8 d were treated with indicated chemo drugs for 3 days. B) AsPC-1 cells were cultured in normoxia, hypoxia 6 h, or hypoxia 7 d and irradiated with X-ray at the indicated dose. The irradiated cells were seeded at clonal density, and the survival fraction ( = number of colony/seeded cells) was calculated. (TIF) [file pone.0098858.s002.tif]

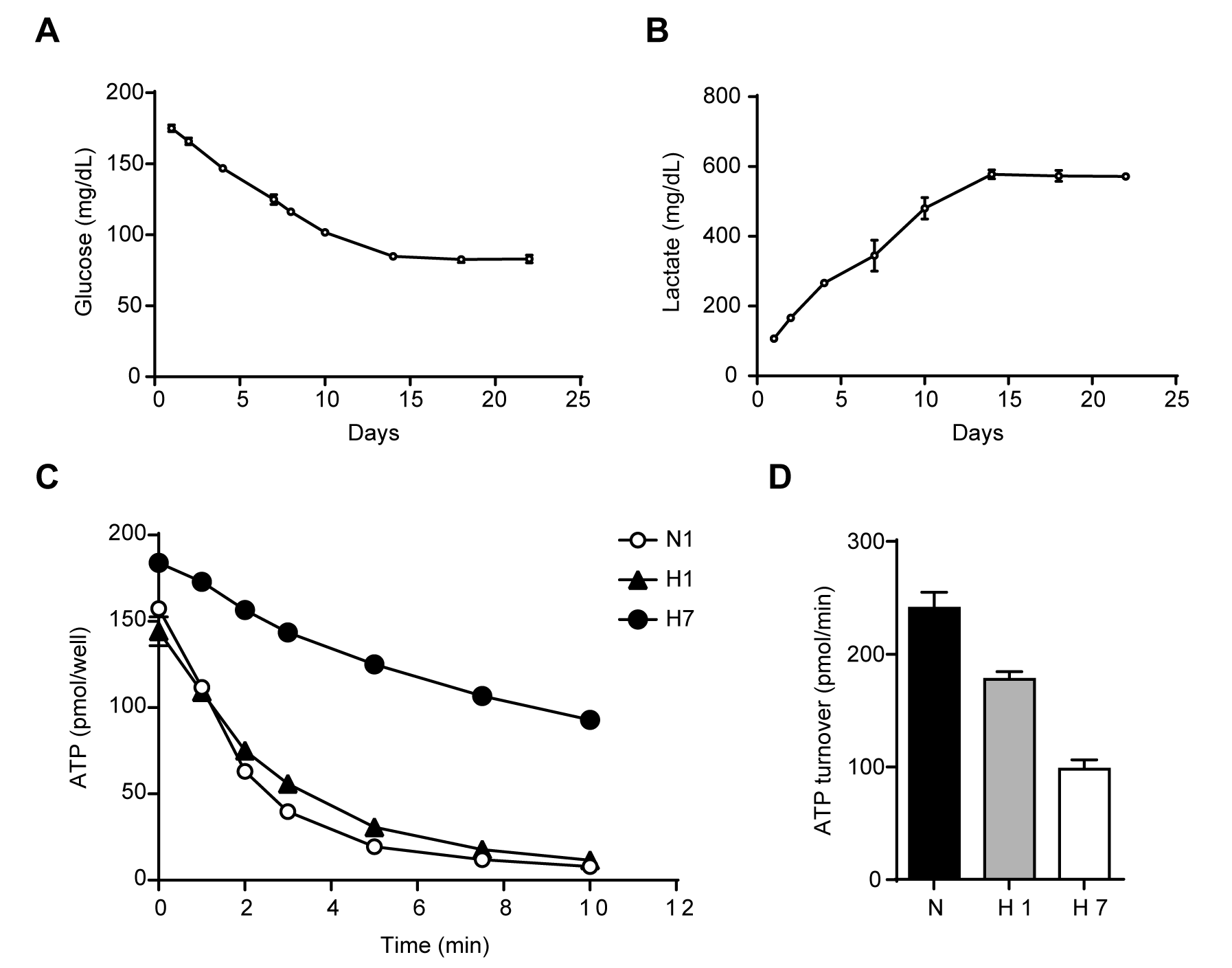

Supplement: Figure S3 — ATP turnover is decreased in chronic hypoxia. Glucose (A) or lactate (B) concentration in conditioned medium of AsPC-1 cells cultured in hypoxia. C) AsPC-1 cells were cultured in a 96-well plate for the indicated periods. Inhibitor cocktail for OXPHOS (KCN, Antimycin A) and glycolysis (2-deoxyglucose) was added at the same time, and the decrease in cellular ATP was measured by Cell Titer Glo. D) ATP turnover was calculated from the slope of the ATP attenuation curve. N1, normoxia 1 day; H1, hypoxia 1 day; H7, hypoxia 7 days. (TIF) [file pone.0098858.s003.tif]

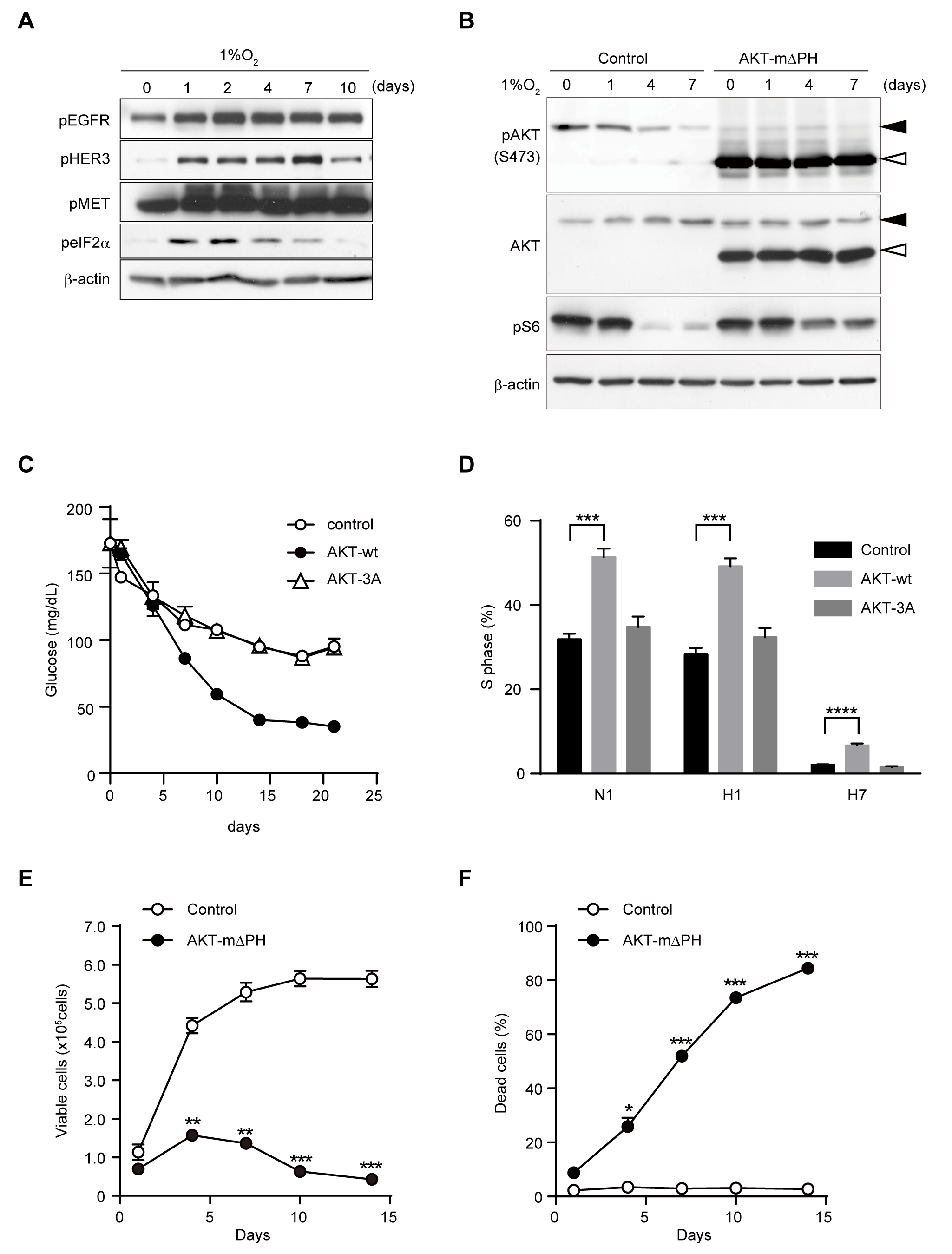

Supplement: Figure S4 — Downregulation of AKT phosphorylation is important for induction of dormant status. A) Western blot of phospho-RTKs or peIF2α in AsPC-1 cells cultured in hypoxia for indicated days. B) Western blot of AKT signaling in AsPC-1 cells expressing control vector or AKT-mΔPH (constitutive active). Black arrowhead indicates endogenous AKT, and white arrowhead indicates AKT-mΔPH. C) Glucose concentration in conditioned medium of AsPC-1 cells cultured in hypoxia. D) Percentage of cells in S phase at normoxia day 1 (N1), hypoxia day 1 (H1), or hypoxia 7 days (H7). Viable cell number (E) and percent cell death (F) of AsPC-1 cells expressing control vector or AKT-mΔPH cultured in hypoxia. *p<0.05, **p<0.01, ***p<0.001, ****p<0.0001. (TIF) [file pone.0098858.s004.tif]

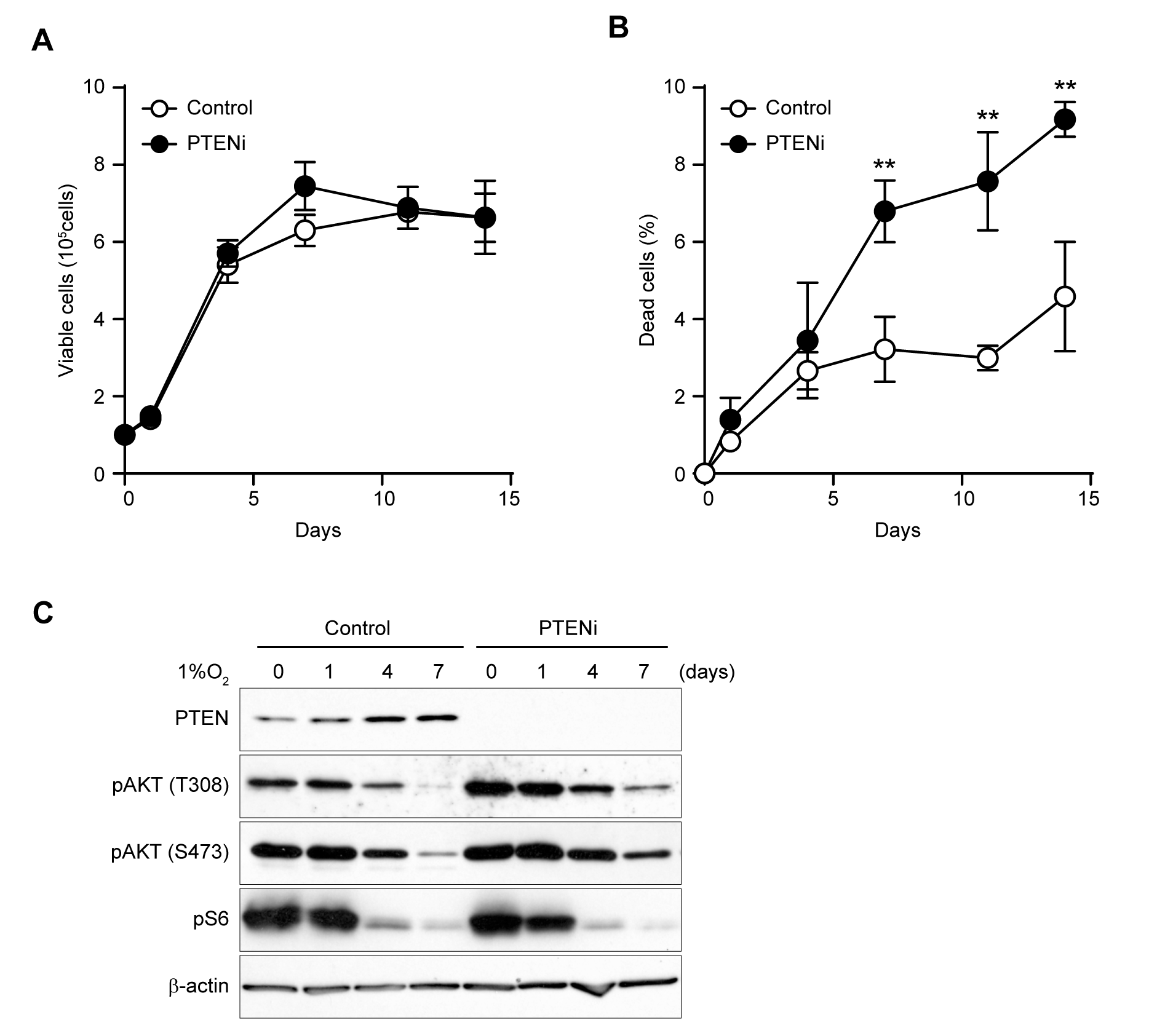

Supplement: Figure S5 — PTEN partially works in the dormant status of AsPC-1 cells. A, B) Viable cell number (A) or percent cell death (B) of AsPC-1 cells expressing control vector or shRNA for PTEN (PTENi) cultured in hypoxia; **p<0.01. C) Western blot of AKT signaling in AsPC-1 cells expressing PTENi. (TIF) [file pone.0098858.s005.tif]

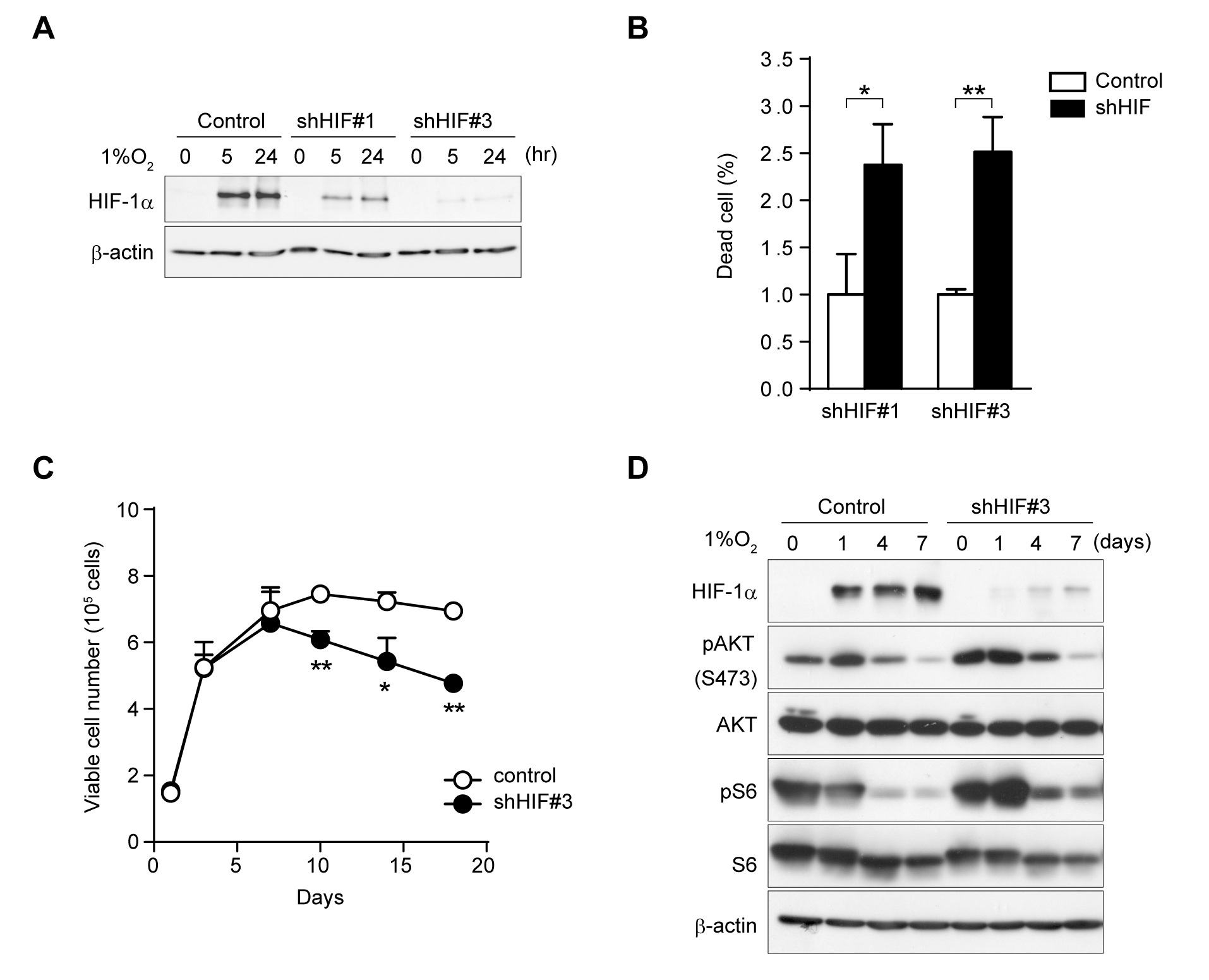

Supplement: Figure S6 — HIF-1α partially contributes to the induction of dormant status in chronic hypoxia. A) Western blot of HIF-1α in AsPC-1 cells expressing control vector or two different clones of shRNA for HIF-1α (shHIF#1 or #3). B) Percent cell death of AsPC-1 cells expressing shHIF#1 or #3 cultured in hypoxia for 10 days. C) Viable cell number of AsPC-1 cells expressing shHIF#3 cultured in hypoxia. D) Western blot of AKT/mTORC1 signaling in AsPC-1 cells expressing shHIF#3. *p<0.05, **p<0.01. (TIF) [file pone.0098858.s006.tif]

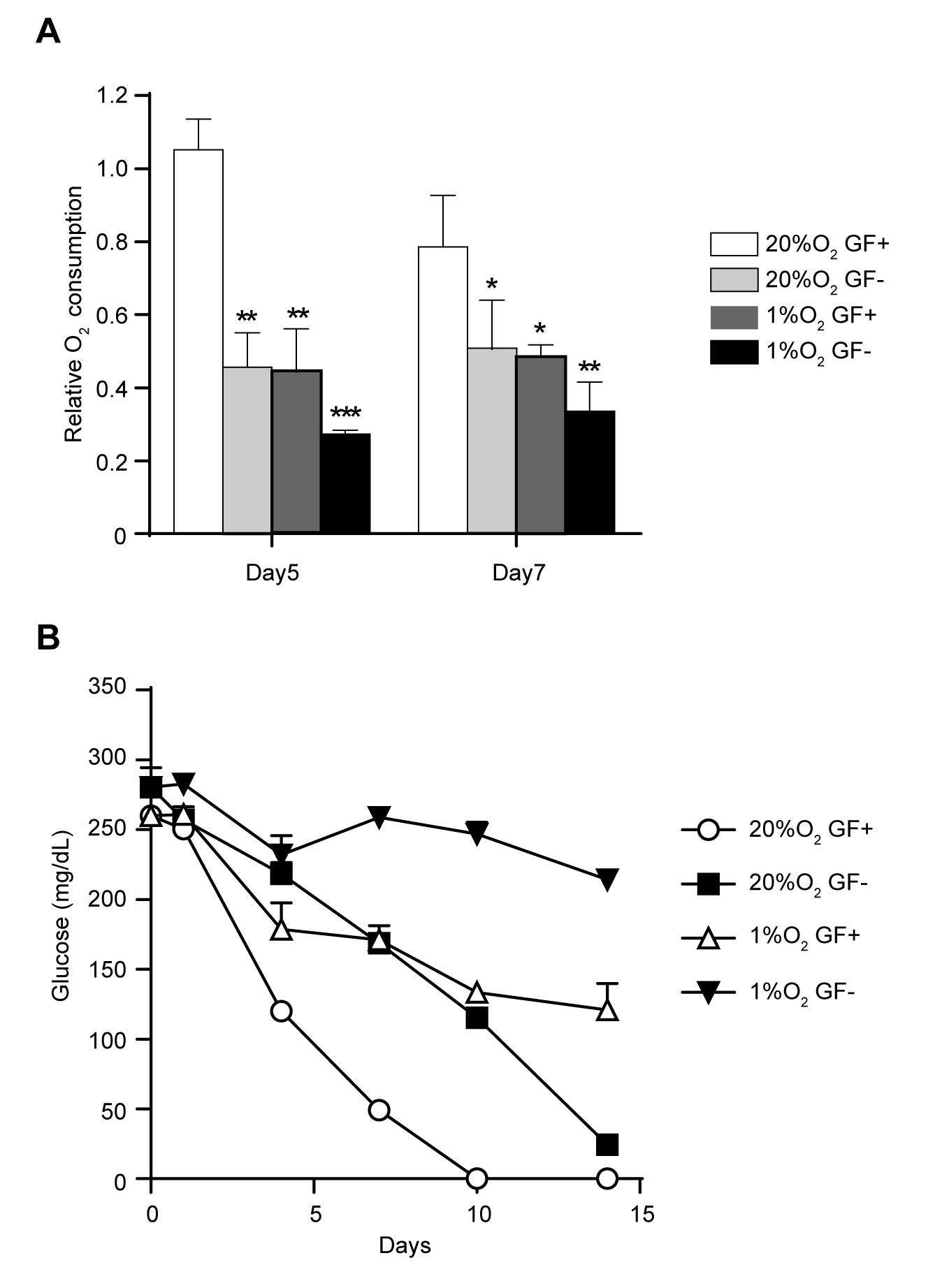

Supplement: Figure S7 — Metabolic processes are suppressed under dormant status in primary colorectal cancer cells. A) Oxygen consumption of C45 CTOS cultured in indicated conditions measured by CRAS; *p<0.05, **p<0.01, ***p<0.001. B) Glucose concentration in culture medium in indicated conditions. (TIF) [file pone.0098858.s007.tif]
