# Supplementary material for: Dormancy of Cancer Cells with Suppression of AKT Activity Contributes to Survival in Chronic Hypoxia
Source: PLoS One. 2014 Jun 6;9(6):e98858. doi: 10.1371/journal.pone.0098858 (PMC4048292; doi:10.1371/journal.pone.0098858)
Supplement: Materials and Methods S1 — (DOC) [file pone.0098858.s008.doc]

**Supplementary Materials and Methods**

**Cells and cell culture**

Pancreatic cancer cell lines MIA PaCa-2 and PANC-1 and colorectal cancer cell lines DLD-1 and COLO320 were obtained from the American Type Culture Collection (ATCC, Rockville, MD). COLO320 cells were cultured in RPMI 1640 medium supplemented with 10% fetal bovine serum (FBS). MIA PaCa-2, PANC-1, and DLD-1 cells were cultured in DMEM supplemented with 10% FBS.

**Reagents**

VP-16, KCN, and 2-deoxyglucose were purchased from Wako Pure Chemical Industries (Osaka, Japan). Antimycin A, 5-Fluorouracil (5-FU), camptothecin, and SN38 were purchased from Sigma-Aldrich (St. Louis, MO).

**Gene silencing**

shRNA targeting HIF-1α (sh#1; 5’-GATGACATGAAAGCACAGA-3’, sh#3; 5’-GACAGTACAGGATGCTTGC-3’), or PTEN (5’-GATCTTGACCAATGGCTAA-3’) was cloned into the retrovirus vector pSuper.retro.puro.

**Oxygen consumption of CTOS**

Oxygen consumption of CTOS was measured by CRAS-1.0 (CLINO, Sendai, Japan).
